# Assessing the Ability of a Large Language Model to Score Free-Text Medical Student Clinical Notes: Quantitative Study

**DOI:** 10.2196/56342

**Published:** 2024-07-25

**Authors:** Harry B Burke, Albert Hoang, Joseph O Lopreiato, Heidi King, Paul Hemmer, Michael Montgomery, Viktoria Gagarin

**Affiliations:** 1 Uniformed Services University of the Health Sciences, Bethesda, MD, 20814, United States, 1 301-938-2212; 2 Defense Health Agency, Falls Church, VA, United States

**Keywords:** medical education, generative artificial intelligence, natural language processing, ChatGPT, generative pretrained transformer, standardized patients, clinical notes, free-text notes, history and physical examination, large language model, LLM, medical student, medical students, clinical information, artificial intelligence, AI, patients, patient, medicine

## Abstract

**Background:**

Teaching medical students the skills required to acquire, interpret, apply, and communicate clinical information is an integral part of medical education. A crucial aspect of this process involves providing students with feedback regarding the quality of their free-text clinical notes.

**Objective:**

The goal of this study was to assess the ability of ChatGPT 3.5, a large language model, to score medical students’ free-text history and physical notes.

**Methods:**

This is a single-institution, retrospective study. Standardized patients learned a prespecified clinical case and, acting as the patient, interacted with medical students. Each student wrote a free-text history and physical note of their interaction. The students’ notes were scored independently by the standardized patients and ChatGPT using a prespecified scoring rubric that consisted of 85 case elements. The measure of accuracy was percent correct.

**Results:**

The study population consisted of 168 first-year medical students. There was a total of 14,280 scores. The ChatGPT incorrect scoring rate was 1.0%, and the standardized patient incorrect scoring rate was 7.2%. The ChatGPT error rate was 86%, lower than the standardized patient error rate. The ChatGPT mean incorrect scoring rate of 12 (SD 11) was significantly lower than the standardized patient mean incorrect scoring rate of 85 (SD 74*; P*=.002).

**Conclusions:**

ChatGPT demonstrated a significantly lower error rate compared to standardized patients. This is the first study to assess the ability of a generative pretrained transformer (GPT) program to score medical students’ standardized patient-based free-text clinical notes. It is expected that, in the near future, large language models will provide real-time feedback to practicing physicians regarding their free-text notes. GPT artificial intelligence programs represent an important advance in medical education and medical practice.

## Introduction

Teaching medical students the skills required to acquire, interpret, apply, and communicate medical information is an integral part of medical education. A crucial aspect of this process involves providing students with feedback regarding the quality of their free-text clinical notes. Various methods have been used to systematically assess clinical notes, notably, QNOTE [1,2], but they depend on human raters. This reliance presents numerous challenges, including rater recruitment and training as well as raters’ availability and inclination to perform reviews. Furthermore, humans are susceptible to biases, fatigue, and misinterpretation.

An attractive innovative alternative to human raters is to use a natural language processing (NLP) program to score student notes. An NLP program is a computer-based algorithm that automatically detects specific meanings in free text. The potential advantages of using an NLP program to grade student notes include the following: it is systematic; it is objective; it avoids human bias, fatigue, and misinterpretation; it is essentially free to run; it can assess any number of notes in seconds; and it can grade notes in real time to provide immediate student feedback.

A new type of NLP program was introduced in November 2022, namely, ChatGPT 3.5 (OpenAI) [3], a large language model (LLM) based on the generative pretrained transformer (GPT) artificial intelligence algorithm. It has achieved a 91.7% score on the United States Medical Licensing Examination (USMLE) style questions [4]. Furthermore, it scored 87.3% on a clinical knowledge test, 91.7% on medical genetics, 89.2% on anatomy, and 92.4% on professional medicine [4]. Its medical-related capabilities include improving clinician empathy [5], responding to patient questions [6], performing differential diagnoses [7], classifying radiology reports [8], writing discharge summaries [9], providing accurate prevention advices to patients [10], and predicting suicide risk [11]. ChatGPT has been compared to human raters in terms of grading short-answer preclerkship medical questions. The ChatGPT-human Spearman correlations for a single assessor ranged from 0.6 to 0.7 [12].

We assessed ChatGPT’s ability to accurately score medical students’ free-text notes on history of present illness, physical examination, and assessment and plan. We compared these scores to standardized patients’ scoring of the clinical notes. We hypothesized that ChatGPT would be more accurate than standardized patients. To our knowledge, this is the first study to assess the ability of a GPT program to score medical students’ standardized, patient-based, clinical free-text notes.

## Methods

### Procedure

This was a single institution, retrospective study. Standardized patients were people who volunteered to interact with medical students to assist in their clinical training. They were trained on a prespecified medical case, and acting as the patient, they interacted with first-year medical students, simulating a patient with that condition. This included responding to clinical questions and undergoing an examination by the medical student. The students documented their interaction with standardized patients in free-text clinical notes. They wrote a chief complaint; history of the present illness; review of systems; physical examination; and differential diagnosis, featuring 3 rank-ordered diagnoses. In addition, they provided their pertinent positives and negatives and suggested follow-up tests. At our medical school, standardized patients provided verbal feedback to students regarding their interaction and scored their students’ notes. They had 7‐10 days to score the student notes and send the results to the course instructor. They did not provide any grading feedback to the students. The advantage of using standardized patients over actual patients for training medical students is that the medical students’ experiences, and therefore, their clinical notes are based on a consistent clinical presentation.

The study case and scoring rubric, “Suzy Whitworth,” were developed by the Association for Standardized Patient Educators and adapted by the Mid-Atlantic Consortium of Clinical Skills Centers in June 2018, with additional formatting edits in January 2019. The standardized patients were trained on this case and its scoring rubric. The case contained 85 scorable elements that were expected to be present in the students’ notes. Three scoring rubric examples were as follows: “Notes chief complaint of shortness of breath (shortness of breath, dyspnea, difficulty breathing, and can’t catch my breath)”; “Notes sudden onset (acute, all of the sudden, and all at once”; and “Notes timing (a few hours ago, this morning, upon awakening, or today).” The rubric combined the 85 scorable elements into 12 classes. ChatGPT and the standardized patients scored as either correct or incorrect each of the 85 elements in the deidentified students’ notes. An error was either an incorrect answer or the absence of an answer. A reviewer checked the standardized patient scoring and the ChatGPT scoring and a second reviewer checked the first reviewer’s scores.

ChatGPT is an LLM based on the GPT artificial intelligence algorithm. It was pretrained on 45 TB of data and it consists of attention, which connects and weights natural language meanings, and an artificial neural network, which organizes and stores the meanings [13]. It accepts natural language input and provides natural language output. For each medical student and for each rubric, the researcher created a new prompt that asked ChatGPT if the rubric’s meaning was contained in the student’s free-text note.

For ChatGPT and standardized patients, the measure of accuracy was the percent correct for each of the 12 categories and across the 12 categories. Student *t* tests (2-tailed) compared the mean error rate across the 12 classes for ChatGPT with the mean error rate across the 12 classes for the standardized patients using the R language (R Project for Statistical Computing) [14].

### Ethical Considerations

Ethical approval was waived as per section 46.104(d) of Code of Federal Regulations, as this was a quality improvement project [15].

## Results

The study population consisted of 168 first-year medical students, the case scoring rubric consisted of 85 elements, resulting in a total of 14,280 scores. There were 4 standardized patients, each working with one-fourth of the students. The incorrect scoring (error) rates for the standardized patients and ChatGPT are shown in Table 1.

**Table 1.** Incorrect scoring rates for ChatGPT and the standardized patients across free-text note categories and across all categories.

Category	Scoring opportunities for the 168 students, n	Standardized patient errors, n (%)	ChatGPT errors, n (%)
Chief complaint	840	135 (16.1)	17 (2.0)
History of present illness	1512	226 (14.9)	35 (2.3)
Review of systems	1008	67 (6.6)	7 (0.7)
Past medical history	1512	43 (2.8)	21 (1.4)
Physical exam	2352	181 (7.7)	25 (1.1)
Diagnosis (pulmonary embolism)	168	3 (1.8)	0 (0)
Pulmonary embolism evidence	2352	182 (7.7)	8 (0.3)
Diagnosis (pneumonia)	168	0 (0)	0 (0)
Pneumonia evidence	1848	66 (3.6)	4 (0.2)
Diagnosis (pneumothorax)	168	0 (0)	7 (4.2)
Pneumothorax evidence	1176	54 (4.6)	5 (0.4)
Diagnostic studies	1008	66 (6.5)	16 (1.6)
Total^a^	14,280	1023 (7.2)	145 (1.0)

a ChatGPT versus standardized patient; *P*=.002.

The category error rates for standardized patients and ChatGPT, respectively, were as follows: chief complaint: 135, 17; history of present illness: 226, 35; review of systems: 67, 7; past medical history: 43, 21; physical examination: 181, 25; first diagnosis: 3, 0; evidence for first diagnosis: 182, 8; second diagnosis: 0, 0; evidence for second diagnosis: 66, 4; third diagnosis: 0, 7; evidence for third diagnosis: 54, 5; and diagnostic studies: 66, 16. The ChatGPT incorrect scoring rate was 1.0%, and the standardized patient incorrect scoring rate was 7.2%. The ChatGPT error rate was 86% lower than the standardized patient error rate. The ChatGPT mean incorrect scoring rate of 12 (SD 11) was significantly lower than the standardized patient mean incorrect scoring rate of 85 (SD 74; *P*=.002).

## Discussion

ChatGPT had a significantly lower error rate compared to standardized patients. This suggests that an LLM can be used to score medical students’ notes.

NLP programs have been used in several medical education settings. Medical education NLPs have been based on keywords, expert systems, statistical algorithms, and combinations of these approaches. DaSilva and Dennick [16] transcribed medical student problem-based verbal learning sessions and used an NLP program to count the frequency of technical words. Zhang et al [17] implemented both a naïve Bayes approach and a supervised support vector machine method to assess resident performance evaluations. Their sentiment accuracies were 0.845 for naïve Bayes and 0.937 for the support vector machine. Spickard et al [18] used an electronic scoring system to detect 25 core clinical problems in medical students’ clinical notes. They achieved a 75% positive predictive value (PPV) on 16 of the 25 problems. Denny et al [19] examined whether students mentioned advance directives or altered mental status in their clinical notes. For advance directives, their sensitivity was 69% and their PPV was 100%, and for mental status, their sensitivity was 100% and their PPV was 93%. Sarker et al [20] used a semisupervised NLP method to assess students’ free-text notes. Their accuracy over 21 cases and 105 notes was a sensitivity of 0.91 and a PPV of 0.87. Two recent papers from the University of Michigan’s Department of Surgery [21,22] assessed resident feedback and competency. Solano et al [21] dichotomized the narrative surgical feedback given to residents into high and low quality and trained a logistic regression model to distinguish between them. Their model achieved a sensitivity of 0.37, a specificity of 0.97, and a receiver operating characteristic (ROC) of 0.86. Otles et al [22] assessed narrative surgical resident feedback using a variety of statistical methods. The support vector machine algorithm achieved the best result with a maximum mean accuracy of 0.64. Abbott [23] studied whether an NLP program could assess the clinical competency committee ratings of residents in terms of language that correlated with the 16 Accreditation Council for Graduate Medical Education Milestones. The ROCs for the 16 milestones ranged from 0.71 to 0.95 and the mean ROC was 0.83. Neves et al [24] examined the ability of RapidMiner Studio, a machine learning program, to assess the quality of attending feedback on resident performance. Their accuracies ranged from 74.4% to 82.2%.

If NLP programs are to be used to automate the grading of students’ notes, they must achieve an acceptable accuracy. Sarker et al [20] suggested that any method of scoring medical notes should achieve an accuracy close to 100%. Regrettably, none of the reported medical education NLPs achieved an acceptable accuracy. In our study, standardized patients also failed to achieve an acceptable accuracy. ChatGPT attained an accuracy close to 100% and is, therefore, suitable for scoring students’ free-text notes.

A potential limitation of this study is that it has been suggested that GPT-based methods have the potential to generate unreliable answers under certain circumstances. We did not find that to be true in our study. Another potential limitation is that, although ChatGPT is free to the public, it has resource requirements. It used 45 TB of data, it has 175 billion parameters, and it runs on supercomputers residing in the cloud. This is a great deal of computing power for student notes. Fortunately, there are open-source GPTs, for example, Meta’s Llama, that can be run on a workstation. We would have liked to examine the standardized patient validity literature, but to our knowledge, no such study exists. Finally, assessing note errors does not directly address clinical reasoning.

An important advantage of LLMs is their ability to provide real-time scoring and feedback on student clinical free-text notes. This immediate assessment offers students a valuable learning opportunity because they can reflect on their performance while the clinical interaction is still fresh in their mind. Another advantage is that the scoring is accurate and objective so students will no longer have to worry about human error and bias. A disadvantage of ChatGPT was that it was time consuming. Fortunately, there are compound GPTs that can perform the entire assessment of all the elements and all the students at once. In terms of clinical reasoning, in the future, we will be asking medical students, as part of their clinical note write-up, to provide their clinical reasoning and we can have a GPT assess the quality of their reasoning.

It should be noted that the use of LLMs to score clinical notes need not be limited to medical students. It is expected that in the near future, GPT-based artificial intelligence NLPs will be applied to provide real-time feedback on free-text clinical notes to practicing physicians.

In conclusion, ChatGPT demonstrated a significantly lower error rate compared to standardized patients. This is the first study to assess the ability of a GPT program to score medical students’ standardized, patient-based, free-text clinical notes. GPT artificial intelligence programs represent an important advance in medical education and medical practice.

## Abbreviations

GPT: generative pretrained transformer
LLM: large language model
NLP: natural language processing
PPV: positive predictive value
ROC: receiver operating characteristic
USMLE: United States Medical Licensing Examination

## References

[1] Burke HB, Hoang A, Becher D (2014). QNOTE: an instrument for measuring the quality of EHR clinical notes. J Am Med Inform Assoc.

[2] Burke HB, Sessums LL, Hoang A (2015). Electronic health records improve clinical note quality. J Am Med Inform Assoc.

[3] OpenAI. ChatGPT.

[4] Singhal K, Tu T, Gottweis J (2023). Towards expert-level medical question answering with large language models. arXiv.

[5] Sharma A, Lin IW, Miner AS, Atkins DC, Althoff T (2023). Human–AI collaboration enables more empathic conversations in text-based peer-to-peer mental health support. Nat Mach Intell.

[6] Ayers JW, Poliak A, Dredze M (2023). Comparing physician and artificial intelligence chatbot responses to patient questions posted to a public social media forum. JAMA Intern Med.

[7] Hirosawa T, Harada Y, Yokose M, Sakamoto T, Kawamura R, Shimizu T (2023). Diagnostic accuracy of differential-diagnosis lists generated by generative pretrained transformer 3 chatbot for clinical vignettes with common chief complaints: a pilot study. Int J Environ Res Public Health.

[8] Olthof AW, Shouche P, Fennema EM (2021). Machine learning based natural language processing of radiology reports in orthopaedic trauma. Comput Methods Programs Biomed.

[9] Patel SB, Lam K (2023). ChatGPT: the future of discharge summaries?. Lancet Digit Health.

[10] Sarraju A, Bruemmer D, Van Iterson E, Cho L, Rodriguez F, Laffin L (2023). Appropriateness of cardiovascular disease prevention recommendations obtained from a popular online chat-based artificial intelligence model. JAMA.

[11] Burkhardt HA, Ding X, Kerbrat A, Comtois KA, Cohen T (2023). From benchmark to bedside: transfer learning from social media to patient-provider text messages for suicide risk prediction. J Am Med Inform Assoc.

[12] Morjaria L, Burns L, Bracken K (2023). Examining the threat of chatgpt to the validity of short answer assessments in an undergraduate medical program. J Med Educ Curric Dev.

[13] Vaswani A, Shazeer N, Parmar N (2023). Attention is all you need. arXiv.

[14] The R Project for Statistical Computing.

[15] Code of Federal Regulations. National Archives.

[16] Da Silva AL, Dennick R (2010). Corpus analysis of problem-based learning transcripts: an exploratory study. Med Educ.

[17] Zhang R, Pakhomov S, Gladding S, Aylward M, Borman-Shoap E, Melton GB (2012). Automated assessment of medical training evaluation text. AMIA Annu Symp Proc.

[18] Spickard A, Ridinger H, Wrenn J (2014). Automatic scoring of medical students’ clinical notes to monitor learning in the workplace. Med Teach.

[19] Denny JC, Spickard A, Speltz PJ, Porier R, Rosenstiel DE, Powers JS (2015). Using natural language processing to provide personalized learning opportunities from trainee clinical notes. J Biomed Inform.

[20] Sarker A, Klein AZ, Mee J, Harik P, Gonzalez-Hernandez G (2019). An interpretable natural language processing system for written medical examination assessment. J Biomed Inform.

[21] Solano QP, Hayward L, Chopra Z (2021). Natural language processing and assessment of resident feedback quality. J Surg Educ.

[22] Ötleş E, Kendrick DE, Solano QP (2021). Using natural language processing to automatically assess feedback quality: findings from 3 surgical residencies. Acad Med.

[23] Abbott KL, George BC, Sandhu G (2021). Natural language processing to estimate clinical competency committee ratings. J Surg Educ.

[24] Neves SE, Chen MJ, Ku CM (2021). Using machine learning to evaluate attending feedback on resident performance. Anesth Analg.

